# Dual-specificity phosphatase (DUSP6) in human glioblastoma: epithelial-to-mesenchymal transition (EMT) involvement

**DOI:** 10.1186/s13104-020-05214-y

**Published:** 2020-08-08

**Authors:** Candida Zuchegna, Erika Di Zazzo, Bruno Moncharmont, Samantha Messina

**Affiliations:** 1 Department of Biology, Federico II University of Naples, 80126 Naples, Italy; 2 Department of Medicine and Health Sciences “V. Tiberio”, University of Molise, 86100 Campobasso, Italy; 3 Department of Science, Roma Tre University, Viale Guglielmo Marconi 446, 00146 Rome, Italy

**Keywords:** Dual-specificity phosphatase (DUSP6), Glioblastoma, Epithelial-to-mesenchymal transition (EMT)

## Abstract

**Objective:**

Glioblastoma (GBM) is the most aggressive and common form of primary brain cancer. Survival is poor and improved treatment options are urgently needed. Dual specificity phosphatase-6 (DUSP6) is actively involved in oncogenesis showing unexpected tumor-promoting properties in human glioblastoma, contributing to the development and expression of the full malignant and invasive phenotype. The purpose of this study was to assess if DUSP6 activates epithelial-to-mesenchymal transition (EMT) in glioblastoma and its connection with the invasive capacity.

**Results:**

We found high levels of transcripts mRNA by qPCR analysis in a panel of primary GBM compared to adult or fetal normal tissues. At translational levels, these data correlate with high protein expression and long half-life values by cycloheximide-chase assay in immunoblot experiments. Next, we demonstrate that DUSP6 gene is involved in epithelial-to-mesenchymal transition (EMT) in GBM by immunoblot characterization of the mesenchymal and epithelial markers. Vimentin, N-Cadherin, E-Cadherin and fibronectin were measured with and without DUSP6 over-expression, and in response to several stimuli such as chemotherapy treatment. In particular, the high levels of vimentin were blunted at increasing doses of cisplatin in condition of DUSP6 over-expression while N-Cadherin contextually increased. Finally, DUSP6 per se increased invasion capacity of GBM. Overall, our data unveil the DUSP6 involvement in invasive mesenchymal-like properties in GBM.

## Introduction

*DUSP6* plays a pro-oncogenic role in cancers such as human glioblastoma, thyroid carcinoma, breast cancer, and acute myeloid leukemia [1–4]. Particularly, DUSP6 is upregulated in human glioblastoma where its overexpression induces reduction in proliferation rate. Cell morphology exhibits a more flattened appearance, lower levels of cellular detachment after stimulation with EGF and an increased propensity to form colonies in soft agar. Surprisingly, mouse xenograft tumors expressing DUSP6 grew significantly faster than controls thus reflecting these changes in cell adhesion and morphology [1]. Moreover, overexpression of DUSP6 has also been identified in a subset of mouse melanoma cell lines, where it is associated with enhanced anchorage-independent growth and invasive capacity [5] and its overexpression in papillary thyroid carcinoma (PTC) is associated with increased cell migration and invasion [2, 6]. Finally, in acute lymphoblastic leukemia (ALL) DUSP6 acts as pro-oncogenic phosphatase in pre-B cell transformation [7].

Glioblastoma (GBM) is the most common and malignant type of primary brain tumor with relevant invasive and resistant properties [8]. The involvement of epithelial-to-mesenchymal transition (EMT) has been extensively investigated in glioblastoma although the real relevance of this program in malignant glioma is still controversial. A number of preclinical studies have been launched to target the process considering the critical role played by EMT on GBM dissemination, resistance to apoptosis and cancer stemness maintenance [9–11]. Of note, DUSP6 is involved in epithelial-to-mesenchymal transition (EMT) in epithelial cancers such as breast and endometrial adenocarcinoma [12, 13]. Moreover, GBMs mesenchymal subtype are characterized by an elevated invasive potential and, of note, the most commonly used glioma cell lines (i.e. U87MG and U251) also present a predominant mesenchymal signature [14] with elevation in mesenchymal markers [15]. The aim of this study was to assess the importance of DUSP6 gene in epithelial-to-mesenchymal transition in GBM in correlation with its invasive capacity.

## Main text

### Methods

#### Cell cultures

Astrocytoma primary (WHO grade IV) GBM#1; GBM#10 etc. were established from tumor specimens of patients and cultured as described [1]. NHA (Normal Human Astrocytes) and NSC (Neural Stem Cells) were purchased from Cambrex (Corporate, NJ 07073, USA) and grown according to the manufacturer’s instructions. Normal Human Astrocytes were used as reference because of the presumed similarity between astrocytes and the cell-of-origin from which glioblastoma develops, both adult (NHA) and foetal (Primary Fetal Normal Neural Stem Cells from SVZ). U87-MG (human GBM–astrocytoma) cell line was purchased from the bank of biological material Interlab Cell Line Collection (Genova, Italy). Human breast cell cultures MCF7, MCF10A and MDA231 cell lines were purchased from the American Type Culture Collection (ATTC, LGC Standards s.r.l, Italy). Cells were cultured at 37 °C in 5% CO_2_ in DMEM with high glucose plus 10% (v/v) fetal bovine serum (FBS, Euroclone, Milan, Italy), penicillin–streptomycin (100 U/mL, Euroclone) and l-glutamine (2 mM, Euroclone), according to manufacturer’s instructions. Primary glioblastomas cell lines (WHO grade IV) were established from tumor specimens of patients and cultured as described [1, 16]. Cells were plated at 80% of confluence on 100 mm dishes and the day after infected with recombinant adenovirus as previously described with the amounts according to the scheme indicated in the figures [16]. In cycloheximide-chase assay experiments the cells were treated with 20 µg/mL CHX in complete medium and then lysed at the indicated times as described in the figures’ legends.

#### Western Blot analysis

Cultured U87-MG and U251-MG cells were washed with PBS and lysed for 15 min in ice-cold Radioimmunoprecipitation (RIPA) buffer (1% Triton X-100, 0.5% deoxycholate-DOC), 0.1% sodium dodecyl sulphate (SDS), 50 mM Tris pH 7.6, 150 mM NaCl, 1 mM phenyl-methyl-sulfonyl fluoride (PMSF), 1 mg/mL aprotinin, leupeptin and pepstatin. Cell lysates were clarified at 12,000 rcf for 30 min at 4 °C and the cytosolic fraction was immediately subjected to protein determination using a Bradford colorimetric assay (Bio-Rad Laboratories Inc., Hercules, CA, USA). DUSP6 was detected with a ‘home-made’ rabbit polyclonal specific antibody against DUSP6 (Lennartson’s lab). Monoclonal anti-α-tubulin as loading control, anti-phospho-ERK and anti-Fibronectin were purchased by Sigma-Aldrich (St Louis, MO, USA). Anti-Vimentin was purchased from Millipore and anti-ERK was purchased from Cell Signaling Technology. Anti N-Cadherin and anti-E-Cadherin were purchased from Santa Cruz Biotechnology. Goat anti-Mouse IgG (H + L) Highly Cross-Adsorbed Secondary Antibody, HRP (A16078) e Goat anti-Rabbit IgG (H + L) Highly Cross-Adsorbed Secondary Antibody, HRP (A16119) Thermofisher Scientific. ECL detection kit from Amersham GE-Healthcare.

#### Real-time quantitative PCR

A quantitative assay for Human *DUSP6* mRNA (seq ref NM_001946.4) expression was established using StepOnePlus™ Real-Time PCR System (Applied Biosystems™) using PowerUp™ SYBR™ Green Master Mix (Applied Biosystems™) using the following program: 95 °C/10 min ×1 cycle; 95 °C/15 s, 63 °C/90 s, ×5 cycles; 95 °C/15 s, 60 °C/90 s, ×35 cycles. All reactions were normalized with the housekeeping gene for 18S. PCR oligo-primers were: Human DUSP6 forward primer 5′-CgAggACCgggACCgCTTCACC-3′ and reverse primer 5′-CCgAgATggggATTTgCTTgTATT-3′ generating a 543 bp fragment; Human 18S forward primer 5′-gACCgATgTATATgCTTgCAgAgT-3′ and reverse primer 5′-ggATCTggAgTTAAACTggTCCAg-3′. The two transcripts of mRNA *DUSP6* were detected by PCR using the following primers: Forward primer 5′-CgAggACCgggACCgCTTCACC-3′; Reverse primer: 5′-AgTTAggggATATgTTggATTTT-3′. The expected size of the fragment was: 758 bp for the transcript variant 1 (NM_001946.4) and 321 bp for the transcript variant 2 (NM_022652.4).

#### Cell invasion assay

Trans-well inserts (Corning^®^ FluoroBlok™ Plate Permeable Support with 8.0 µm Colored PET Membrane) for 24-well plates were used. Inserts were coated with Geltrex™ Matrix (Gibco by Life Technologies). U87MG cells were transduced with TRK or DUSP6 expressing adenoviral vectors and 5 × 10^4^ cells were seeded in serum-free Dulbecco’s modified Eagle’s medium in the upper chamber on the top of the matrigel. Dulbecco’s modified Eagle’s medium 10% FBS (600 μL) was dispensed in the lower chamber as a chemoattractant. After 48 h the apparatus was washed with PBS and cells that did not migrate were removed with a cotton swab, then inserts were fixed (3.7% paraformaldehyde for 20 min at room temperature. The results were quantified by counting all the cells of the inserts in duplicate from two independent experiments using the 10× objective. Data were tested for normal distribution of variables using the Shapiro–Wilks test and statistical significance between groups was determined using Student’s t-test.

### Results

**Fig. 1:**
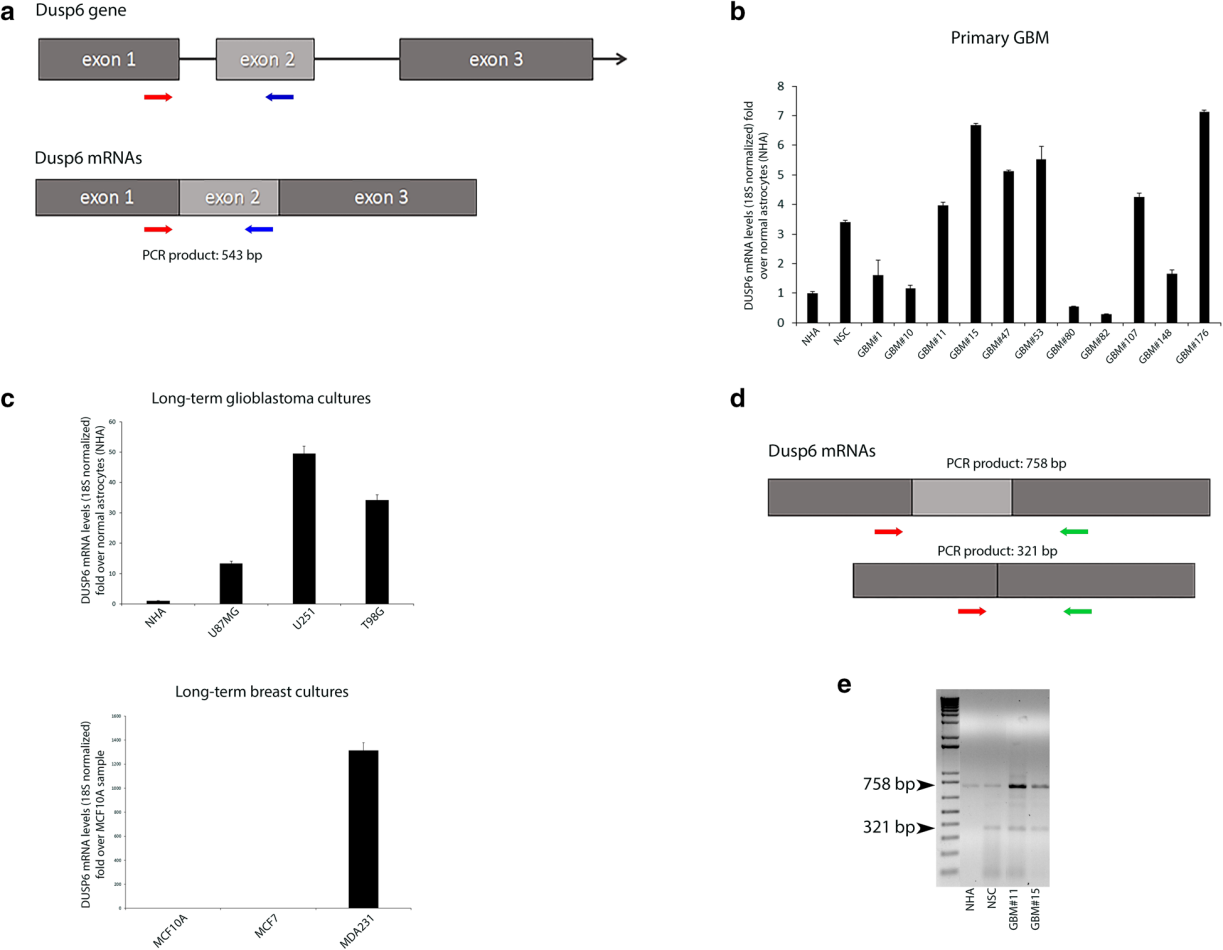
DUSP6 mRNA is over-expressed in human glioblastomas. **a** Schematic grey boxes denote protein coding region of *DUSP6* gene and colored arrows represent the approximate locations of the primers’ annealing sites in *DUSP6* gene. **b** Bar diagram shows relative quantification of total DUSP6 mRNA across a panel of human glioblastoma samples (grade IV astrocytoma). Normal Human Astrocytes (NHA) and Neural Stem Cells (NSC) were used as controls because of the presumed similarity between astrocytes and the cell-of-origin from which glioblastoma develops, both adult (NHA) and foetal (NSC). Relative expression was normalized to housekeeping gene 18S expression. **c** Upper panel: relative *DUSP6* mRNA expression quantified by qPCR on human glioblastoma cultures U251MG, U87MG and T98G. Lower panel: human breast cell cultures MCF7, MCF10A and MDA231 were assayed as positive controls. **d** Qualitative PCR for *DUSP6* mRNA in primary glioblastomas. Expression of different-size transcripts was detected using primers shown in the upper panel. Schematic denotes black/grey boxes exons and the relative location of the forward and reverse primer annealing sites in *DUSP6* mRNA gene. Ethidium stained agarose gel of end-point products from the different amplicons DUSP6 using Normal Human astrocytes (NHA), Neural Stem Cells (NCS) and two samples of primary glioblastoma mRNA. The two alternative transcripts of *DUSP6* mRNA are shown (transcript variant 1; NM_001946.4, and transcript variant 2 NM_022652.4)

Quantitative transcriptional analysis of *DUSP6* was assessed by RT-qPCR measuring high mRNA levels in a panel of twenty primary glioblastomas (GBM, WHO grade 4) (Fig. 1b) by quantifying the mRNA fold-induction over Normal Human Astrocytes (NHA) and Neural Stem Cells (NSC) which specify distinct glioblastoma subtype [17–20]. We measured increased expression in primary samples (approximatively sevenfold enrichment in GBM#15, GBM#53 and GBM#176) compared to controls. In addition, human long-term cultures U87MG, U251MG and T98G displayed high mRNA levels compared to primary samples of GBM (their values differ by several orders of magnitude) (Fig. 1c). Furthermore, mRNA levels in breast cancers lines MCF-7, MCF-10 were undetectable but not in MDA-MB-231, a model for triple-negative breast cancer, expressing aberrantly high levels (50- and 6000-fold enrichment) (Fig. 1c). Interestingly, the gene contains three distinct introns (Fig. 1a) producing short- and long-PCR products (sized 758 bp and 321 bp respectively) with a marked prevalence of the long-transcript in primary glioblastomas (personal observation), i.e. samples GBM#11 and GBM#15 (Fig. 1e).

**Fig. 2:**
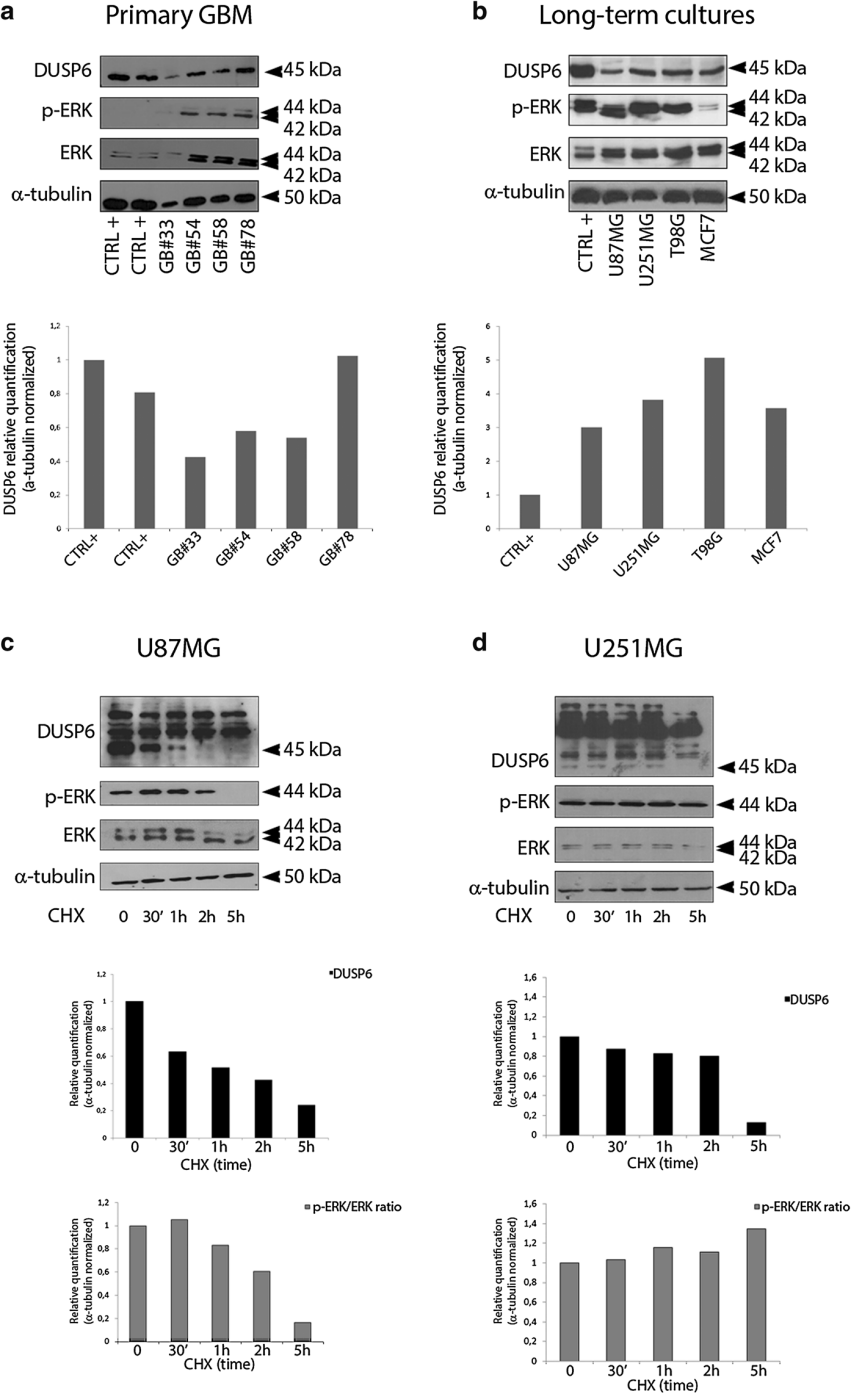
DUSP6 protein is over-expressed in human glioblastomas. **a** Western Blot analysis of *DUSP6*, p-ERK and ERK in primary GBM samples. In both panels CTRL+ indicates positive immune-reactive control obtained with U87MG infected with the adenovirus DUSP6. **b** Western Blot analysis of *DUSP6*, p-ERK and ERK in U87MG, U251MG, T98G and MCF7 cell lines. The corresponding bar graphs show relative expression of proteins normalized to α-tubulin. **c**, **d** Post-translational regulation of *DUSP6* protein levels- U87MG and U251MG cell lines were treated with cycloheximide (CHX, 20 µg/mL) for the time indicated and the decay of the target proteins over time was determined by Western Blot with specific antibodies. The corresponding bar graphs show relative expression of proteins normalized to α-tubulin

Further, we used cycloheximide-chase assay after single-time point western blotting as a measurement of half-life endogenous DUSP6 protein (Fig. 2). Unstimulated primary GBM (Panel a) and long-term cultures (Panel b) show high protein levels in both primary and long-term glioblastoma as assayed by semi-quantitative Western blot analysis (Fig. 2a, b). The ERK/MAPK cascade activation was sustained in both primary and long-term cultures U87MG, U251 and T98G, while over-expression adenovirus-mediated (CTRL+) completely abrogate p-ERK and ERK ½ signals (Fig. 2a). De novo protein synthesis was measured by time-course cycloheximide (CHX) experiment in both cell lines (Fig. 2c, d). We found that acutely inhibiting protein synthesis diminished DUSP6 diminished expression in both U87MG and U251MG but did so with different kinetics. In contrast to previously published data [21] we report long half-life in both cell lines U87MG and U251 (respectively more than 1 h and up to 5 h). These results agree with the stable endogenous protein exerting oncogenic properties in cancers.

**Fig. 3:**
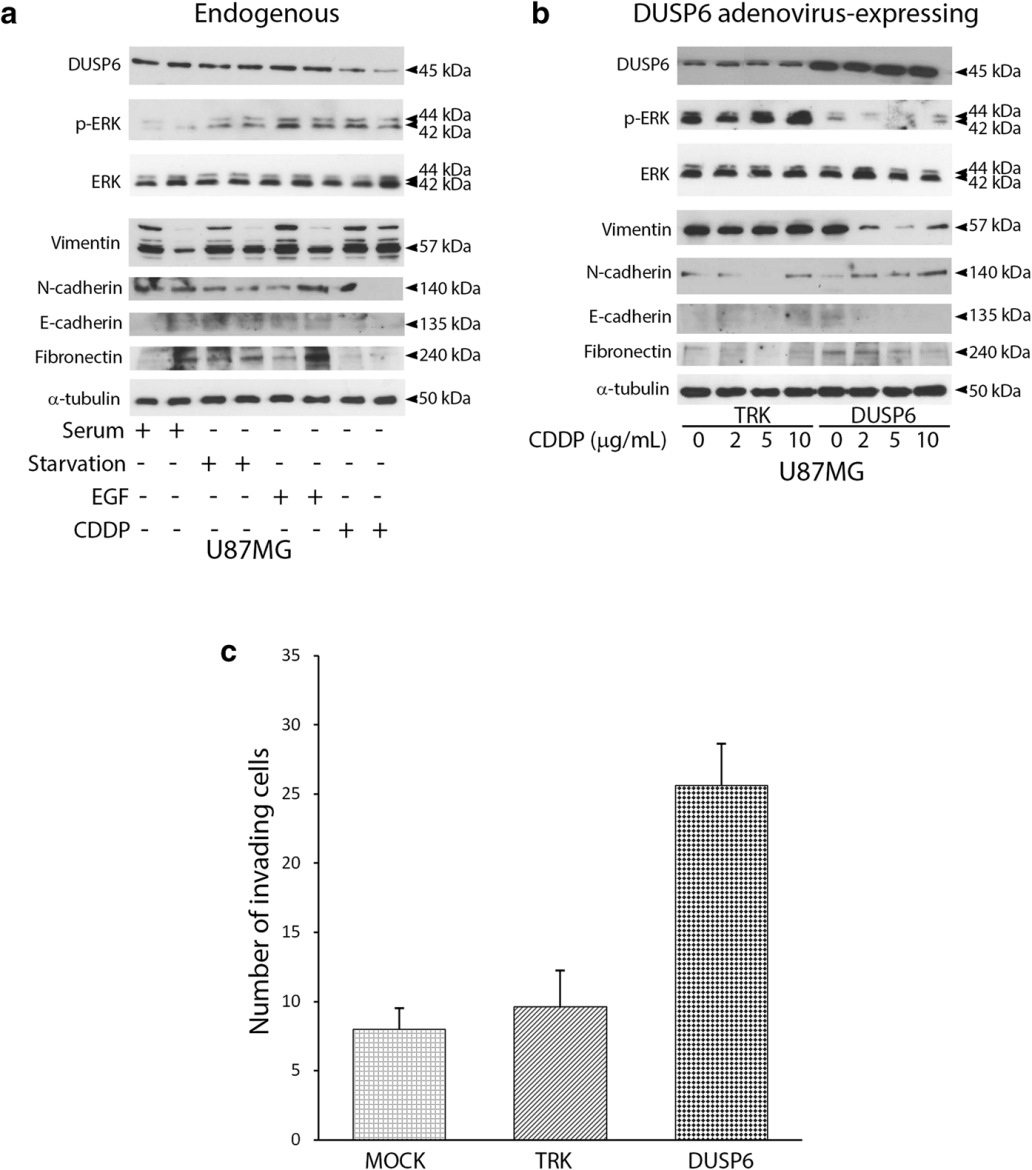
Differential regulation of epithelial and mesenchymal markers by DUSP6. **a** Immunoblot analysis of proteins DUSP6, p-ERK, ERK, vimentin, N-Cadherin, E-Cadherin, fibronectin in long-term cultures U87MG treated with different stimuli (serum, starvation, EGF 100 ng/mL, cisplatin (CDDP) 5 µg/mL) for 5 h (left lane) or 24 h (right lane). **b** Immunoblot analysis of mesenchymal and epithelial markers in U87MG adenovirus-expressing DUSP6 compared to TRK (empty vector). The cells were treated with increasing doses of cisplatin (CDDP) at different concentrations (respectively 0, 2, 5, 10 μg/mL) and protein expression assayed by western blot on total lysates at 5 h. **c** Invasion assay. Bar graph reports the mean of total number of invaded cells per field compared to control (MOCK i.e. U87MG not infected). U87MG cells were transduced with adenovirus-expressing DUSP6 or control vector (TRK i.e. U87MG infected with empty vector) and seeded on matrigel-coated trans-well inserts. The experiment was performed for 48 h and the invading cells were counted (10× objective) in nine randomly chosen microscopic fields per trans-well. Data are presented as mean ± S.D. from two independent experiments performed in duplicate

Then, we examined the expression of mesenchymal markers associated with EMT in glioblastoma cultures, in naïve condition and in DUSP6-overexpression. Firstly, we assayed protein endogenous levels of Vimentin, N-Cadherin, E-Cadherin, Fibronectin in U87MG upon several stimuli (serum addition, serum deprivation, EGF and cisplatin) by Western Blot with specific antibodies (Fig. 3a). Immunoblot analysis for p-ERK/ERK ratio shows different kinetics of ERK/MAPK kinases phosphorylation upon EGF and cisplatin (CDDP) treatments.

Interestingly, cisplatin treatment reduces the amount of N-Cadherin, E-Cadherin and Fibronectin, but not Vimentin (Fig. 3a). N-Cadherin and Fibronectin were slightly up-regulated in adenovirus-mediated DUSP6 overexpression (Fig. 3b). Otherwise, the epithelial marker E-Cadherin was almost absent in both naïve and adenoviral-expressing cells. Next, in DUSP6-overexpressing U87MG cells increasing doses of cisplatin (ranging from 2 to 10 µL/mL) affected the EMT markers in opposite fashion. Vimentin protein expression is completely blunted upon treatment whereas high levels are shown in adenoviral-expressing control vector (CTRL) and in adenoviral-expressing DUSP6 untreated (lane 5) (Fig. 3b). Finally, we assayed invasive ability of both naïve glioblastoma cells compared to DUSP6-adenoviral-expressing U87MG cells by Trans-well invasion assay (see “Methods”). We here report that DUSP6 increased the invasion capacity of the glioma U87MG cells (Fig. 3c) compared to MOCK cells (naïve U87MG) and adenoviral empty vector as negative control (TRK).

### Discussion

This study was designed to explore DUSP6 involvement in epithelial-to-mesenchymal transition in glioblastoma. We previously showed that DUSP6 is upregulated in human glioblastoma and in vitro adenovirus-mediated overexpression results in a transformed phenotype [1]. Here, we extended our transcriptional analysis to include a new set of primary cell cultures of glioblastoma and long-term cultures. By using qRT-PCR, we found high levels of mRNA DUSP6 across the glioblastoma samples. Moreover, we assayed breast cancer cell lines and found that the mesenchymal MDA-MB-231 showed the highest levels of mRNA expression compared to normal epithelial MCF10 and MCF7 cell lines. Interestingly, DUSP6 is involved in maintaining the mesenchymal state in breast cancer [12]. Moreover, here we report high protein expression and long half-life values by cycloheximide-chase assay in long-term cultures U87MG and U251, in line with previous data on fibroblasts [21]. We demonstrate that DUSP6 gene is involved in epithelial-to-mesenchymal transition (EMT) in GBM. Whereas we clearly show that DUSP6 per se increases invasion capacity of GBM, the evidence on epithelial-to-mesenchymal transition in GBM does not lead to a firm conclusion. We report immunoblot characterization of the mesenchymal and epithelial markers with and without DUSP6 over-expression. Vimentin is clearly down-regulated in cisplatin-treated over-expressing DUSP6 cells but not in absence of cisplatin. N-Cadherin is up-regulated in cisplatin-treated over-expressing DUSP6 whereas it was down-regulated in naïve cells. In addition, our data on cadherin switch are in line with the inconsistency of the literature, with some reports showing that GBM do not express E-Cadherin, but others showing the occurrence of an E- to N-Cadherin switch. More importantly, our data show that cisplatin treatment clearly downregulates Fibronectin and both Cadherins while Vimentin underwent no change in naïve cells (in absence of DUSP6 over-expression). Notably, classical cadherin switch, which is widely accepted as an EMT hallmark in carcinomas, is a controversial matter in GBM [22, 23].

Of note, it was found that N-cadherin expression is inversely correlated with the invasive behavior of GBM, and its ectopical expression reduces cell migration and restores polarity in GBM cells [24, 25]. Conversely, lower expression of N-Cadherin was recognized in a panel of GBM primary samples at mRNA and protein levels [26].

Recently, association of the EMT transition with chemo-resistance has been reported [27]. Our results clearly show that mesenchymal markers are down-regulated by cisplatin in naïve cells, except for vimentin. This is in line with vimentin increased levels in resistant GBM cultures compared to parental ones [28]. Moreover, conflicting results are reported on DUSP6’ role in chemotherapy-resistance in epithelial cancers [29, 30]. Here we report the first observation of EMT markers in response to a drug used in chemotherapy during DUSP6 over-expression. Particularly, the high levels of vimentin were blunted at increasing doses of cisplatin in a condition of DUSP6 over-expression while N-Cadherin contextually increased. These data have implications on chemotherapy response in glioblastoma treatment.

## Limitations

Limitations of this study include: (i) limited number of low-passage, serum-free cell lines cultured from patient tumor tissue (GBM# in the text) (ii) the poorly-representative cell line models (U87MG and U251MG), which are not exhaustive model for glioblastoma multiforme molecular subtypes (classical, pro-neural and mesenchymal) [31]. Further, this study reports only an in vitro characterization based on western blot analysis of the classical mesenchymal markers (Additional file 1) and, from the point-of-view of phenotypic characterization, the invasion assay does not exhaustively demonstrate that DUSP6 activates EMT in GBM by enhancing its invasive properties.

## Supplementary information

**Additional file 1: Figure S1.** Uncropped versions of the western blot used in this manuscript (corresponding to Fig. 2) - Original gels of Western Blot analysis of Fig. 2 panels A and B: immune-reactive bands corresponding to specific antibodies against *DUSP6*, p-ERK and ERK and α-tubulin as specified in Methods section. Original gels of Western Blot analysis of Fig. 2 panels C and D: immune-reactive bands corresponding to specific antibodies against *DUSP6*, p-ERK and ERK and α-tubulin as specified in Methods section. **Figure S2.** Uncropped versions of the western blot used in this manuscript (corresponding to Fig. 3) - Original gels of Western Blot analysis of Fig. 3 panels A and B: immune-reactive bands corresponding to specific antibodies against DUSP6, p-ERK, ERK, Vimentin, N-Cadherin, E-Cadherin, Fibronectin as specified in Methods section.

## Data Availability

All data presented or analyzed in this study are included in this article.

## Abbreviations

DUSP6: Dual-specificity phosphatase 6
CHX: Cycloheximide
ERK1/2: Extracellular regulated kinases
EMT: Epithelial-to-mesenchymal transition
ALL: Acute lymphoblastic leukaemia
GBM: Glioblastoma

## References

[1] Messina S, Frati L, Leonetti C, Zuchegna C, Di Zazzo E, Calogero A, Porcellini A (2011). Dual-specificity phosphatase DUSP6 has tumor-promoting properties in human glioblastomas. Oncogene.

[2] Degl’Innocenti D, Romeo P, Tarantino E, Sensi M, Cassinelli G, Catalano V, Lanzi C, Perrone F, Pilotti S, Seregni E (2013). DUSP6/MKP3 is overexpressed in papillary and poorly differentiated thyroid carcinoma and contributes to neoplastic properties of thyroid cancer cells. Endocr Relat Cancer.

[3] Song HM, Wu CY, Wei CK, Li DF, Hua KY, Song JL (2015). Silencing of DUSP6 gene by RNAi-mediation inhibits proliferation and growth in MDA-MB-231 breast cancer cells: an in vitro study. Int J Clin Exp Med.

[4] Arora D, Köthe S, van den Eijnden M, van Huijsduijnen RH, Heidel F, Fischer T (2012). Expression of protein-tyrosine phosphatases in Acute Myeloid Leukemia cells: FLT3 ITD sustains high levels of DUSP6 expression. Cell Commun Signal.

[5] Li W, Song L, Ritchie AM, Melton DW (2012). Increased levels of DUSP6 phosphatase stimulate tumorigenesis in a molecularly distinct melanoma subtype. Pigment Cell Melanoma Res.

[6] Lee JU, Huang S, Lee MH, Lee SE, Ryu MJ, Kim SJ, Kim YK, Kim SY, Joung KH, Kim JM (2012). Dual specificity phosphatase 6 as a predictor of invasiveness in papillary thyroid cancer. Eur J Endocrinol.

[7] Shojaee S, Caeser R, Buchner M, Park E, Swaminathan S, Hurtz C, Geng H, Chan LN, Klemm L, Hofmann WK (2015). Erk negative feedback control enables Pre-B cell transformation and represents a therapeutic target in acute lymphoblastic leukemia. Cancer Cell.

[8] Kahlert UD, Joseph JV, Kruyt FAE (2017). EMT- and MET-related processes in nonepithelial tumors: importance for disease progression, prognosis, and therapeutic opportunities. Mol Oncol.

[9] Iser IC, Pereira MB, Lenz G, Wink MR (2017). The epithelial-to-mesenchymal transition-like process in glioblastoma: an updated systematic review and in silico investigation. Med Res Rev.

[10] Mehta S, Lo Cascio C (2018). Developmentally regulated signaling pathways in glioma invasion. Cell Mol Life Sci.

[11] Kubelt C, Hattermann K, Sebens S, Mehdorn HM, Held-feindt J (2015). Epithelial-to-mesenchymal transition in paired human primary and recurrent glioblastomas. Int J Oncol.

[12] Boulding T, Wu F, McCuaig R, Dunn J, Sutton CR, Hardy K, Tu W, Bullman A, Yip D, Dahlstrom JE (2016). Differential roles for DUSP family members in epithelial-to-mesenchymal transition and cancer stem cell regulation in breast cancer. PLoS ONE.

[13] Fan MJ, Liang SM, He PJ, Zhao XB, Li MJ, Geng F (2019). Dusp6 inhibits epithelial-mesenchymal transition in endometrial adenocarcinoma via ERK signaling pathway. Radiol Oncol.

[14] Behnan J, Finocchiaro G, Hanna G (2019). The landscape of the mesenchymal signature in brain tumours. Brain.

[15] Lu KV, Chang JP, Parachoniak CA, Pandika MM, Aghi MK, Meyronet D, Isachenko N, Fouse SD, Phillips JJ, Cheresh DA, Park M, Bergers G (2012). VEGF inhibits tumor cell invasion and mesenchymal transition through a MET/VEGFR2 complex. Cancer Cell.

[16] Messina S, Leonetti C, De Gregorio G, Affatigato V, Ragona G, Frati L, Zupi G, Santoni A, Porcellini A (2004). Ras inhibition amplifies cisplatin sensitivity of human glioblastoma. Biochem Biophys Res Commun.

[17] Lindberg N, Kastemar M, Olofsson T, Smits A, Uhrbom L (2009). Oligodendrocyte progenitor cells can act as cell of origin for experimental glioma. Oncogene.

[18] Liu C, Sage JC, Miller MR, Verhaak RGW, Vogel H, Foreman O, Bronson RT, Nishiyama A (2011). Mosaic analysis with double markers (MADM) reveals tumor cell-of-origin in glioma. Cell.

[19] Alcantara Llaguno SR, Wang Z, Sun D, Chen J, Xu J, Kim E, Hatanpaa KJ, Raisanen JM, Burns DK, Johnson J (2015). Adult lineage restricted CNS progenitors specify distinct glioblastoma subtypes. Cancer Cell.

[20] Alcantara L, Chen J, Kwon C, Jackson EL, Li Y, Burns DK, Alvarez-buylla A, Parada LF (2009). Malignant astrocytomas originate from neural stem/progenitor cells in a somatic tumor suppressor mouse model. Cancer Cell.

[21] Marchetti S, Gimond C, Chambard JC, Touboul T, Roux D, Pouysségur J, Pagès G (2005). Extracellular signal-regulated kinases phosphorylate mitogen-activated protein kinase phosphatase 3/DUSP6 at serines 159 and 197, two sites critical for its proteasomal degradation. Mol Cell Biol.

[22] Siebzehnrubl FA, Silver DJ, Tugertimur B, Deleyrolle LP, Siebzehnrubl D, Sarkisian MR, Devers KG, Yachnis AT, Kupper MD, Neal D (2013). The ZEB1 pathway links glioblastoma initiation, invasion and chemoresistance. EMBO Mol Med.

[23] Lewis-Tuffin LJ, Rodriguez F, Giannini C, Scheithauer B, Necela BM (2010). Misregulated E-cadherin expression associated with an aggressive brain tumor phenotype. PLoS ONE.

[24] Asano K, Duntsch CD, Zhou Q, Weimar JD, Bordelon D, Robertson JH, Pourmotabbed T (2004). Correlation of N-cadherin expression in high grade gliomas with tissue invasion. J Neurooncol.

[25] Camand E, Peglion F, Osmani N, Sanson M, Etienne-Manneville S (2012). N-cadherin expression level modulates integrin-mediated polarity and strongly impacts on the speed and directionality of glial cell migration. J Cell Sci.

[26] Musumeci G, Magro G, Cardile V, Coco M, Marzagalli R, Castrogiovanni P, Imbesi R, Graziano AC, Barone F, Di Rosa M, Castorina S, Castorina A (2015). Characterization of matrix metalloproteinase-2 and -9, ADAM-10 and N-cadherin expression in human glioblastoma multiforme. Cell Tissue Res.

[27] Ashrafizadeh M, Zarrabi A, Hushmandi K, Kalantari M, Mohammadinejad R, Javaheri T, Sethi G (2020). Association of the epithelial-mesenchymal transition (EMT) with cisplatin resistance. Int J Mol Sci.

[28] Liao H, Bai Y, Qiu S, Zheng L, Huang L, Liu T, Wang X, Liu Y, Xu N, Yan X, Guo H (2015). MiR-203 downregulation is responsible for chemoresistance in human glioblastoma by promoting epithelial-mesenchymal transition via SNAI2. Oncotarget.

[29] Gao Y, Li H, Han Q, Li Y, Wang T, Huang C, Mao Y, Wang X, Zhang Q, Tian J, Irwin DM, Tan H, Guo H (2020). Overexpression of DUSP6 enhances chemotherapy-resistance of ovarian epithelial cancer by regulating the ERK signaling pathway. J Cancer.

[30] James NE, Beffa L, Oliver MT, Borgstadt AD, Emerson JB, Chichester CO, Yano N, Freiman RN, DiSilvestro PA, Ribeiro JR (2019). Inhibition of DUSP6 sensitizes ovarian cancer cells to chemotherapeutic agents via regulation of ERK signaling response genes. Oncotarget.

[31] Clark MJ, Homer N, O’Connor BD, Chen Z, Eskin A, Lee H, Merriman B, Nelson SF (2010). U87MG decoded: the genomic sequence of a cytogenetically aberrant human cancer cell line. PLoS Genet.

