# Supplementary material for: Dual-specificity phosphatase (DUSP6) in human glioblastoma: epithelial-to-mesenchymal transition (EMT) involvement
Source: BMC Res Notes. 2020 Aug 8;13:374. doi: 10.1186/s13104-020-05214-y (PMC7414695; doi:10.1186/s13104-020-05214-y)

Figure S1: Uncropped versions of the Western Blots used in this manuscript
(corresponding to the Figure 2)

WB:
Figure 2
Panel A

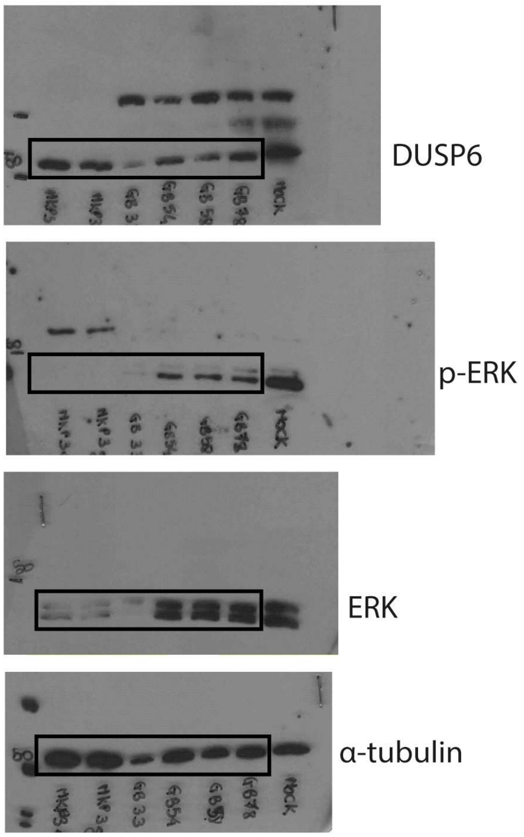

WB:
Figure 2
Panel B

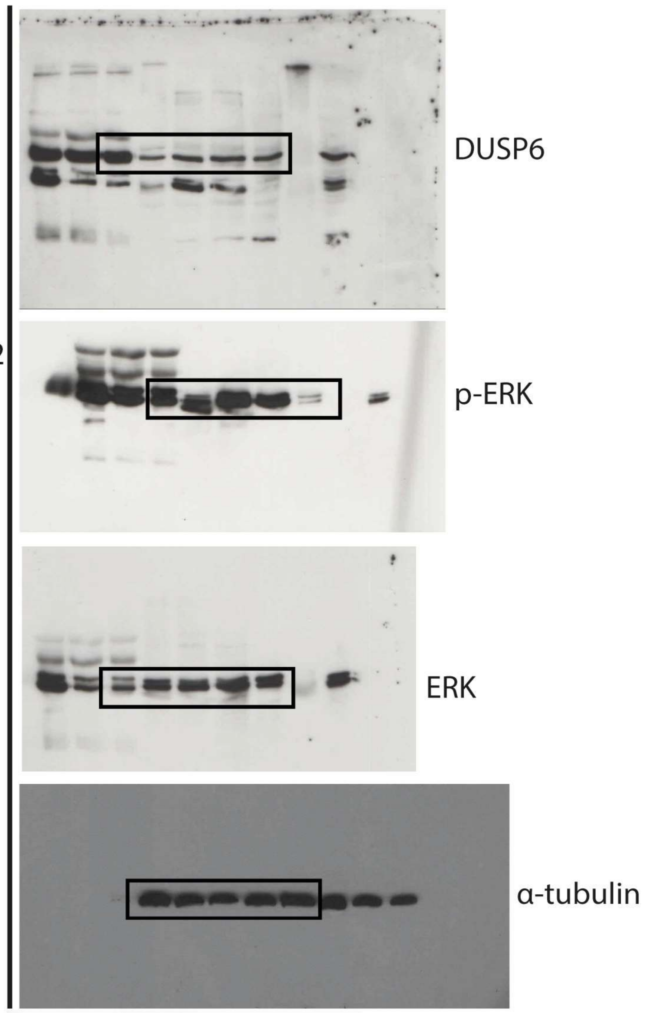

WB:
Figure 2
Panel C

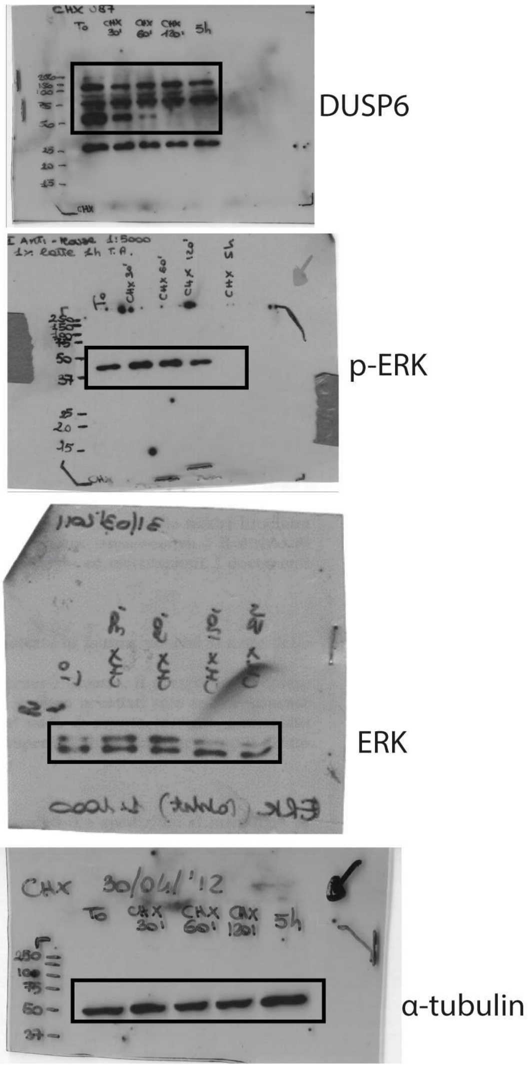

WB:
Figure 2
Panel D

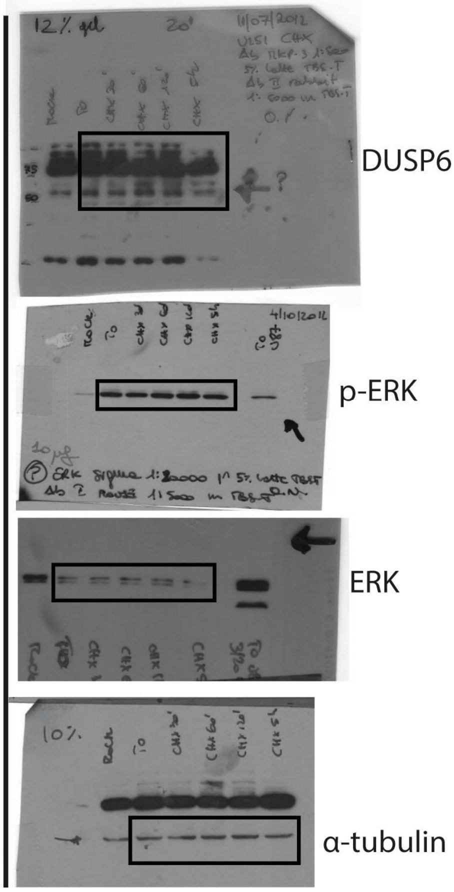

Figure S2: Uncropped versions of the Western Blots used in this manuscript (corresponding to the Figure 3)

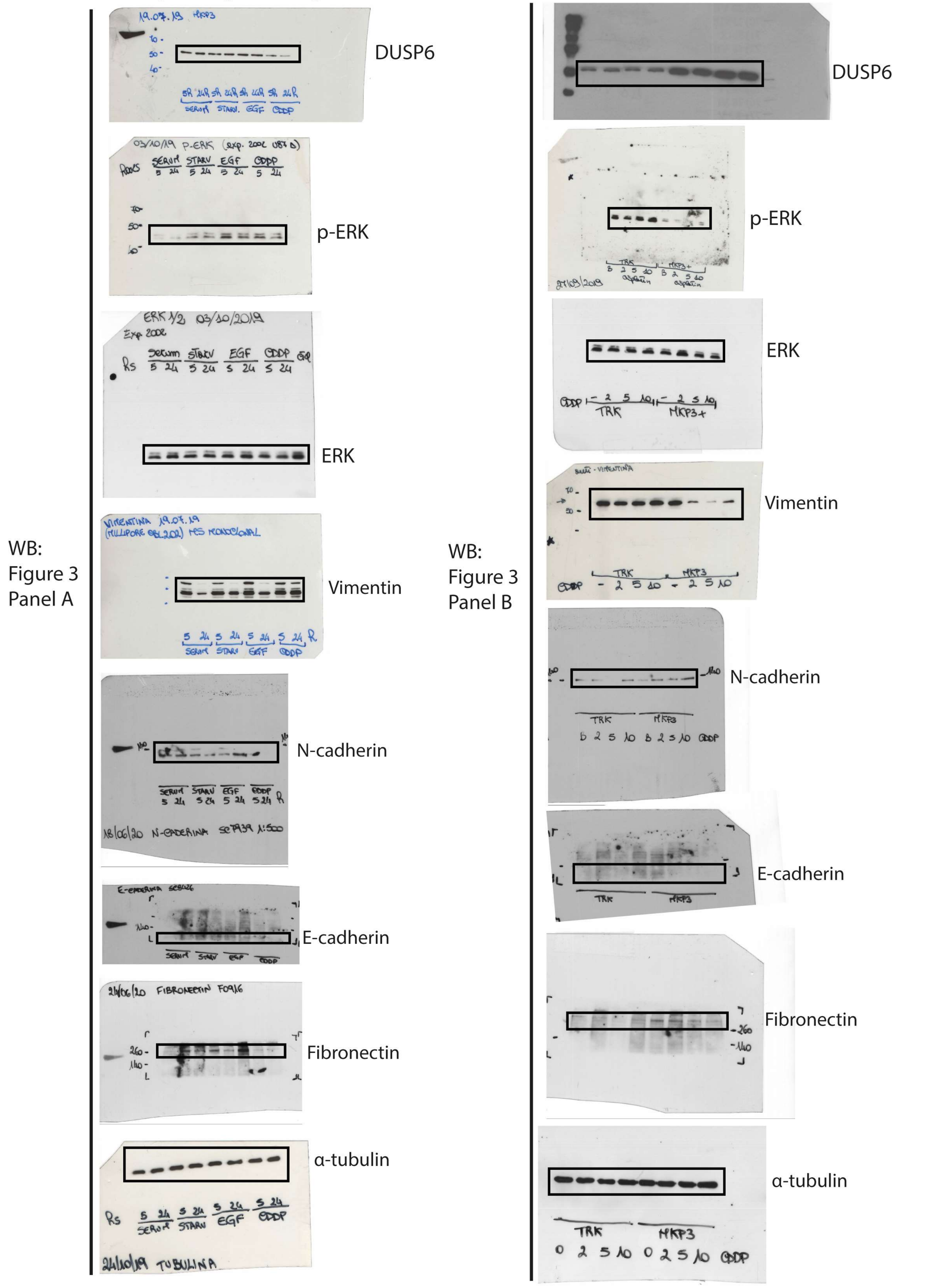

Supplement: Supplementary file 1 — Additional file 1: Figure S1. Uncropped versions of the western blot used in this manuscript (corresponding to Fig. 2) - Original gels of Western Blot analysis of Fig. 2 panels A and B: immune-reactive bands corresponding to specific antibodies against DUSP6, p-ERK and ERK and α-tubulin as specified in Methods section. Original gels of Western Blot analysis of Fig. 2 panels C and D: immune-reactive bands corresponding to specific antibodies against DUSP6, p-ERK and ERK and α-tubulin as specified in Methods section. Figure S2. Uncropped versions of the western blot used in this manuscript (corresponding to Fig. 3) - Original gels of Western Blot analysis of Fig. 3 panels A and B: immune-reactive bands corresponding to specific antibodies against DUSP6, p-ERK, ERK, Vimentin, N-Cadherin, E-Cadherin, Fibronectin as specified in Methods section. [file 13104_2020_5214_MOESM1_ESM.pdf]
